# A Promising New Therapy of Oral Ixazomib Without Rituximab for Waldenstrom Macroglobulinemia

**DOI:** 10.4274/tjh.galenos.2020.2020.0521

**Published:** 2021-02-25

**Authors:** Wanlu Ma, Jiawei Zhao, Lu Zhang

**Affiliations:** 1 Peking Union Medical College Hospital, Chinese Academy of Medical Sciences and Peking Union Medical College, Department of Endocrinology, Beijing, China; 2 Peking Union Medical College Hospital, Chinese Academy of Medical Sciences and Peking Union Medical College, Department of Rheumatology, Beijing, China; 3 Peking Union Medical College Hospital, Chinese Academy of Medical Sciences and Peking Union Medical College, Department of Hematology, Beijing, China

**Keywords:** Waldenstrom macroglobulinemia, Rituximab-induced thrombocytopenia, Ixazomib

## To the Editor,

**Figure 1:**
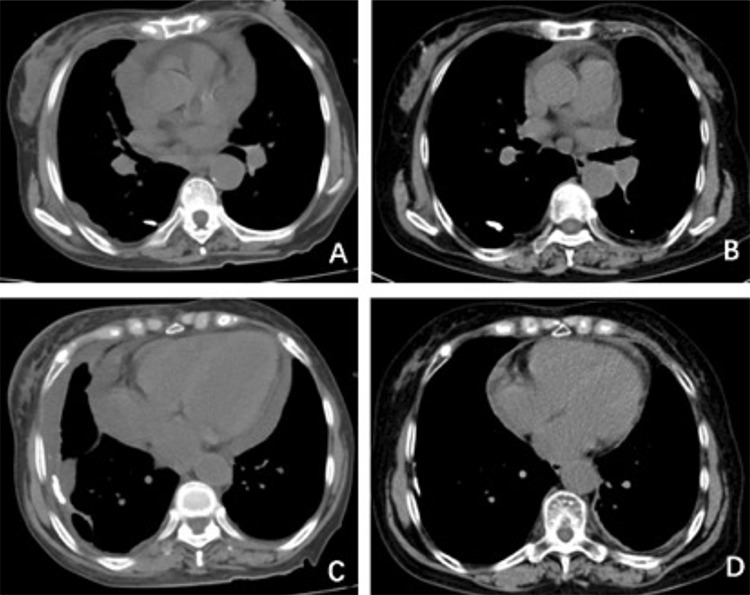
Computed tomography (CT) scans showed multiple enlarged lymph nodes in the mediastinum and pericardial and pleural effusion before treatment **(A, C)**. CT scan showed complete disappearance of enlarged lymph nodes and pericardial or pleural effusion after treatment **(B, D)**.

A 73-year-old woman was admitted with complaints of low fever, fatigue, shortness of breath, and edema of the lower extremities. Hemoglobin (Hb) and platelets (PLTs) were 76-93 g/L (normal range: 110-160 g/L) and 75-103x10^9^/L (100-300x10^9^/L), respectively. Serum protein electrophoresis indicated that M protein and immunoglobulin M were elevated to 8.6 g/L and 11.9 g/L (0.4-2.3 g/L). Blood immunofixation electrophoresis also exhibited positive immunoglobulin M (IgM)κ. Computed tomography (CT) scans revealed multiple enlarged lymph nodes in the mediastinum, mesentery, neck, underarms, and groin with pericardial and pleural effusion and splenomegaly (Figures 1A and 1C). A bone marrow smear revealed 7.5% lymphocytic plasma cells, whose immune phenotypes were consistent with Waldenstrom macroglobulinemia (WM). Furthermore, L265P mutation in *MYD88* was detected in the bone marrow and pleural effusion. Accordingly, WM was diagnosed and the RCD regimen (rituximab, dexamethasone, cyclophosphamide) was given for 2 cycles, but her PLTs quickly decreased to 15x10^9^/L with petechiae during the infusion of rituximab, probably due to rituximab-induced thrombocytopenia [1]. Two weeks later, PLTs gradually increased to 80x10^9^/L, but Hb remained at 54 g/L and M protein did not decrease. A CT scan did not show any reduction of pericardial or pleural effusion. Meanwhile, a new-onset femoral neck fracture prevented her from coming to the hospital for a bortezomib-based regimen and economic status restricted her from usage of ibrutinib and ICD (ixazomib, dexamethasone, cyclophosphamide). Therefore, an oral treatment regimen was given for 1 cycle followed by 5 cycles of ID regimen (ixazomib, dexamethasone) due to neutropenia possibly induced by cyclophosphamide. Strikingly, Hb rose back to 125 g/L and PLTs remained stable at 155x10^9^/L. M protein fell back to 0.6 g/L. A CT scan showed normal size of multiple lymph nodes and absence of pericardial and pleural effusion (Figures 1B and 1D). The patient achieved very good partial response (VGPR) after 6 cycles of ixazomib-based regimen and remained in VGPR without maintenance for 1 year.

WM is a rare indolent hematologic disorder sometimes requiring treatment due to IgM-secreting lymphoplasmacytic cells in the bone marrow and other organs [2,3]. L265P mutation of *MYD88* is present in more than 90% of cases [4]. Primary therapy options include anti-CD20 monoclonal antibodies, mainly rituximab. However, proteasome inhibitors (bortezomib, carfilzomib, ixazomib) are playing a greater role both in primary therapy and as salvage options. Bortezomib has been extensively studied and proved effective as a single agent [5], but bortezomib-associated neuropathy and toxicity limit its widespread use. Carfilzomib combined with dexamethasone has also been reported to be effective for WM [6]. However, the role of ixazomib, another oral proteasome inhibitor, has not been well illuminated. Although the IDR regimen (ixazomib, rituximab, dexamethasone) was suggested to be effective, well tolerated, and neuropathy-sparing in a prospective phase II study with 96% overall response rate in 26 symptomatic patients with WM [3], the efficacy of ixazomib-based regimens without rituximab has not been reported before. Considering that rituximab alone could be effective in WM patients (52% overall response rate) [7] and oral ixazomib might be especially useful in the outpatient setting with less economic burden and no need for continuous treatment compared to oral ibrutinib and bortezomib, it would be helpful to delineate the efficacy of ixazomib-based oral treatment in WM patients.

Herein, we have reported the first clinical case of a patient with WM who could not tolerate rituximab due to rituximab-induced thrombocytopenia and responded well to oral ixazomib administered at home after a femoral neck fracture. Ixazomib may be considered for those not tolerating rituximab or bortezomib or those who cannot receive them for other reasons. Whether maintenance therapy adds benefits to the prognosis remains controversial [3]. Our patient remained in VGPR without maintenance therapy for a year. Further evidence concerning the benefits of ixazomib maintenance is needed. 

In conclusion, our case highlights the strengths of ixazomib-based regimens without rituximab in patients with WM. As an oral proteasome inhibitor, the unique role of ixazomib in treating WM awaits further investigations.
